# Hydrothermal Effect on Ramie-Fiber-Reinforced Polymer Composite Plates: Water Uptake and Mechanical Properties

**DOI:** 10.3390/polym15143066

**Published:** 2023-07-17

**Authors:** Anni Wang, Peng Yin, Xiaogang Liu, Guijun Xian

**Affiliations:** 1 Research Institute of Urbanization and Urban Safety, School of Civil and Resource Engineering, University of Science and Technology Beijing, Beijing 100083, China; 2 Key Lab of Smart Prevention and Mitigation of Civil Engineering Disasters of the Ministry of Industry and Information Technology, Harbin Institute of Technology, Harbin 150090, China; 3 Key Lab of Structures Dynamic Behavior and Control of the Ministry of Education Harbin Institute of Technology, Harbin 150090, China; 4 School of Civil Engineering, Harbin Institute of Technology, Harbin 150090, China

**Keywords:** ramie-fiber-reinforced polymer composites, hydrothermal environment, water absorption, degradation of mechanical properties

## Abstract

Ramie-fiber-reinforced polymer composites (RFRP) have the advantages of low price and low energy consumption, but they have high hydrophilicity due to their special chemical composition. In order to study the effect of water absorption on the performance degradation of RFRP in a hydrothermal environment, the authors prepared RFRP sheets by compression molding. Manufactured composites were exposed to a hydrothermal environment with a temperature of 40 °C and a humidity of 50% RH, 85% RH and 98% RH to study the water absorption and diffusion, mechanical properties (tensile properties, flexural properties and shear properties) of the RFRP, and their mechanical properties after drying. The research shows that the equilibrium moisture absorption rate of RFRP is mainly affected by the ambient humidity. The moisture absorption and diffusion of ramie-fiber-reinforced polymer composites (RFRP) in a hydrothermal environment conform to Fick’s law. Before reaching the moisture absorption equilibrium (1~2 weeks), the mechanical properties decline rapidly, and then tend to be flat, and the mechanical properties of the RFRP decrease significantly with the increase in humidity; the water molecules reduce the interfacial bonding performance and the modulus degradation degree of RFRP in the hydrothermal environment is greater than that of strength. After the samples were completely dried, the mechanical properties of the RFRP rebounded greatly, but less than the initial value, and the hydrothermal environment produced irreversible changes to the substrates.

## 1. Introduction

Fiber-reinforced polymer composites (FRP) are structural composite materials that have been widely used in aerospace, machinery, building structures and other fields [1]. Common FRP include composite materials such as glass fiber, aramid fiber and carbon fiber. However, due to the increasing awareness of environmental protection around the world, natural-fiber-reinforced polymers (NFRP) are gradually being valued [2]. NFRP mainly has the following advantages [3,4].

(1) Low density: the relative density of natural fiber is approximately 1.5, while that of glass fiber is 2.6.
(2) The price is low: the cost per kilogram of natural fiber is USD 0.9~1.08 less than that of glass fiber.
(3) Low energy consumption: to produce the same product, the use of NFRP consumes less non-renewable energy than other materials.

Natural fibers are mainly composed of cell walls and cell lumens. Cellulose, hemicellulose and lignin are the main components of cell walls, and the substances contained in the cell lumens are collectively called pectin [5]. Cellulose is a white polysaccharide macromolecule compound with the molecular formula (C_6_H_10_O_5_)n, where n is the number of β-glucose monomers in the cellulosic polysaccharide macromolecule, generally 2000~2500 [6]. Hydrogen bonds hold these molecules together into the microfibrils of the filaments. The physical and chemical properties of ramie fibers are mainly determined by cellulose, which plays a decisive role in the corresponding RFRP, including moisture absorption properties, hydrolysis properties after moisture absorption, and corresponding mechanical properties [7,8]. Ramie fiber has a large cellulose (hydrophilic group) content and contains a large number of hydroxyl groups [9]. The polarity of cellulose and the swelling and dissolving properties of cellulose are related to the hydroxyl groups on the cellulose macromolecules, and the elongated cavities in the fibers and the cracks on the surface determine that it has strong hygroscopicity [10].

The hygroscopicity of natural plant fibers is much greater than that of carbon fibers, glass fibers, etc. [11]. Moisture absorption leads to fiber swelling, forming holes and microcracks at the fiber–resin interface, thereby reducing the transmission of effective stress [12]. In addition, compared with FRP such as carbon fiber and glass fiber, the mechanical properties of NFRP decrease significantly after moisture absorption, and both the strength and modulus decrease [13]. Therefore, in order to better apply NFRP in practical engineering, it is necessary to understand the hygroscopic properties of NFRP and the influence of moisture absorption on mechanical properties, and find effective ways to reduce hygroscopicity.

Ana Espert et al. [14] studied the hygrothermal aging properties of natural wood cellulose fabric-reinforced polypropylene composites, and found that the water absorption and diffusion rate of the material was affected by temperature in accordance with the Arrhenius regularity, and the mechanical properties were also greatly affected by the hygrothermal environment, with a relatively large degradation. Abdul Moudood et al. found that the mechanical properties of flax/bio-epoxy composites are clearly degraded by water ageing, and water incurred more severe effects on the flexural properties of the composites [15]. Ming Cai et al. studied the effects of hydrothermal aging on the tensile and interlaminar shear characteristics of the composites. Long-term hydrothermal aging was found to weaken interlaminar shear characteristics of the composites, causing degradation/deformation of the flax fibers and degradation of the phenolic matrix [16]. The mechanical properties of NFRP are susceptible to be changed by moisture/water absorption. In most cases, the moisture/water uptake can cause a notable decrease in tensile and flexural properties due to plasticization and the weakening of the fiber–matrix interface [17]. Researchers have also studied the improvement of durability, such as improving the interface and interlayer properties of composite materials through surface treatment [18] and designing functionally graded materials to improve their durability [19].

When NFRP are used in practical projects, they must be exposed to high-temperature and high-humidity environments. High temperature and heat transfer is an important issue in construction of composite structures [20]. High-humidity environments will cause hygroscopicity, and water molecules will cause reversible and irreversible changes in the properties of NFRP [21]. The so-called reversible change means that after the NFRP absorbs moisture, the physical properties of the natural fiber and resin temporarily decrease, and when the free water is removed, the performance can return to the original state. The so-called irreversible change means that the performance will not recover after water is removed. It is generally believed that the reversible change is the result of the natural fiber absorbing free water and the water plasticizing the resin, and the irreversible change is the degradation and degradation of the plant fiber and resin structure and the interface layer structure by water. The force transmission mechanism of composite materials is determined by the fiber–resin interface. Studies have shown that the impact of water molecules on the fiber–resin interface after the composite material absorbs moisture mainly includes the following aspects [22,23,24]

(1) Resin swelling after moisture absorption leads to interface debonding and destruction.

After the NFRP absorbs moisture, the resin matrix swells accordingly [25]. Due to the different strain changes in the fiber resin after moisture absorption, the plant fibers wrapped in the resin generate a shear stress. When the swelling shear stress is greater than the bonding stress of the interface, the interface is debonded [26].

(2) The osmotic pressure of water in the pores leads to debonding and failure of the fiber–resin interface.

Due to the poor wettability of the natural fiber and the resin matrix, the resin matrix cannot spread well on the surface of the natural fiber, resulting in a certain gap and the fiber–resin interface containing micropores. When water enters the material, the water gathers in the micropores, and some impurities in the resin base dissolve in the water, forming a concentration difference between the inside and outside. As time goes by, the concentration of the aqueous solution in these micropores increases continuously. Once the osmotic pressure exceeds, the NFRP will suffer from interfacial debonding failure.

Ramie, an ancient plant with a long history, is being researched because of its vigorous growth, high nutritional value, environmental conservation and medicine. It can be cultivated in versatile conditions including tropical, subtropical and temperate regions [27]. Besides, ramie is cultivated as a major economic crop in China: 500,000 tons of fiber is produced per year, accounting for approximately 96% of global production [28]. Phenolic resin has excellent high-temperature resistance, and can maintain structural integrity and stability at higher temperatures. At the same time, phenolic resin has good compatibility and bonding strength with ramie fiber. In addition, phenolic resin is the earliest industrialized synthetic resin. Due to the easy availability of raw materials and convenient synthesis, the resin can meet various industrial requirements after curing, and has been widely used in industry. Therefore, this paper takes ramie fiber as the research object to study the durability of ramie-fiber-reinforced phenolic resin composite in hydrothermal environment.

In summary, ramie-fiber-reinforced polymer composite (RFRP) easily absorbs water in a hydrothermal environment, resulting in the degradation of its mechanical properties and dimensional instability, which affects the engineering application of RFRP. In this paper, manufactured composites were exposed to a hydrothermal environment with a temperature of 40 °C and a humidity of 50% RH, 85% RH and 98% RH to study the water absorption and diffusion, mechanical properties (tensile properties, flexural properties and shear properties) of the RFRP, and their mechanical properties after drying. Therefore, it is of great significance to systematically study the hygroscopic properties of RFRP in hydrothermal environments and the degradation regularity and mechanisms of their mechanical properties to promote the safe and reliable application of RFRP in actual engineering structures.

## 2. Experimental

### 2.1. Materials and Sample Preparation

#### 2.1.1. Materials

Ramie fiber fabric was provided by the Beijing Institute of Aeronautical Material (Beijing, China). The warp/weft density was 64 × 66 and the areal density was 140 g/m^2^. Ramie fiber is a woven bidirectional fabric, in which the radial fiber content is higher than the weft fiber content. Radial fibers are mainly used for load bearing, and weft fibers are used for fixing. The phenolic resin with brand name F51 was purchased from Nantong Xingchen Synthetic Material Co., Ltd. (Nantong City, China). The model number is 1052451101, and its solid content is determined to be approximately 46.1 wt.% according to the GJB 1059.1-90 standard. The resin curing temperature and period were 130 °C and 2 h, respectively.

#### 2.1.2. RFRP Sample Fabrication

Ramie fiber fabric-reinforced phenolic resin (RFRP) was prepared from ramie fiber fabric and liquid phenolic resin. First, the phenolic resin was diluted to 80% solids with acetone. The ramie fabric is then impregnated with diluted phenolic resin using a brush. Finally, place the impregnated fabric in a dark and ventilated room for at least 2 days until the acetone in the resin evaporates completely. The ramie prepreg containing phenolic resin can then be used in thermocompression molding process. 12 pieces of prepreg (25 × 25 cm) were molded in a thermocompression molding machine (XLB model, Dongya Nanxing Machinery Co., Ltd., Qingdao, China) at 130 °C and 10 MPa pressure for 2 h, and remove it until it cools to room temperature. The RFRP is a laminate prepared by laying up bidirectional ramie fabric and the fiber direction of each layer is consistent. The thickness of the prepared RFRP plate was 2.77 mm, and the volume of the ramie fabric was approximately 40.4%.

#### 2.1.3. Humid Environments

According to ASTM D5229/D5229M, select ambient humidity: 50% RH, 85% RH and 95% RH. The experimental humidity environments were prepared by saturated salt solution. The types of salt solution and their corresponding relative humidity are shown in Table 1. Use a plastic box to hold a certain amount of corresponding saturated saline solution, seal it with polyvinyl chloride film, and place it in a constant-temperature water tank to form a constant-temperature and -humidity environment. The hygrometer was used to test three kinds of hydrothermal environments, and the results showed that all the devices met the set humidity and temperature requirements, indicating that the use of saturated salt solution and constant-temperature water tank can meet the environmental requirements of the experiment. The other environmental factors are completely excluded in the experiment.

#### 2.1.4. Moisture Absorption and Desorption Tests

Prior to exposure to various humid conditions, all samples (25 × 25 × 2.77 mm^3^) for water absorption and desorption tests were oven-dried for 48 h at 60 °C in accordance with ASTM D5229/5229M. After drying, each sample was weighed, and the mass was designated as its initial mass, M_0_. During exposure, samples were periodically weighed according to the test plan (i.e., 2 h, 4 h, 8 h, 1 day, 2 days, 4 days, 1 week, 2 weeks, 1 month, 3 months, and 6 months) and mass changes were recorded. After exposure for 90 days, samples were taken out and dried at 60 °C in an oven. The mass change in the samples was recorded as a function of drying time. For each condition, ten samples were repeatedly tested; mean value and standard deviation were reported.

#### 2.1.5. The Mechanical Properties Test

Tensile test for RFRP samples was conducted according to ASTM D3039. The RFRP plates were cut into 250 × 15 × 2.77 mm^3^. The test was carried out at load speed of 5 mm/min with extensometer gage length of 50 mm. The short beam shear strength (SBS) of the RFRP samples was tested according to ASTM D2344/D2344M. The size of the samples was 16.6 × 5.5 × 2.77 mm^3^. The testing span was 11 mm and the test speed rate was 1 mm/min. The flexural performance test of the specimens was carried out according to the ASTM D790 standard. The size of the bending sample is 56.8 × 12.7 × 2.77 mm^3^, the thickness t of the sample is approximately 2.75 mm, and the span is 44 mm; the test speed is 1.17 mm/min.

## 3. Results and Discussion

### 3.1. Moisture Absorption and Desorption

**Figure 1:**
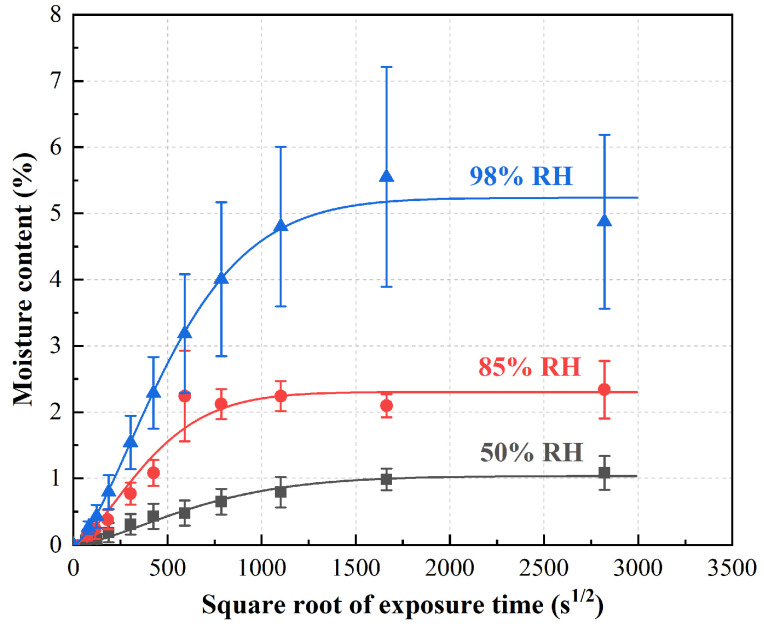
Moisture uptake curves of ramie fiber fabric-reinforced phenolic resin under three kinds of humidity at 40 °C.

The plot of mass increase versus square root of exposure time (Figure 1) shows the moisture absorption rate of RFRP exposed to three hydrothermal environments (50% RH, 85% RH and 95% RH) at 40 °C for 6 months. Under the environment of 50% RH and 85% RH, the moisture absorption increases linearly at the beginning of the exposure period and then tends to be stable with the prolongation of the exposure time, which conforms to Fick’s law. However, at 98% RH, except for the last test value, the trend of the moisture absorption curve is similar to that at 50% RH and 85% RH. The last measured value of the moisture absorption curve decreases with increasing exposure time, deviating from the saturated moisture content. Therefore, Fick’s law is not suitable for the whole moisture absorption process of RFRP immersed in the 98% RH. Under three different humidity conditions, the time for RFRP to reach equilibrium water absorption was different. RFRP reaches equilibrium water absorption more slowly at 50% RH than at 85% RH. In the 98% RH humidity environment, because it does not conform to Fick’s law, its regularity is inconsistent with that of 50% RH and 85% RH. The initial linear region is caused by the diffusion of water molecules in the micro-gap between the polymer chains [29]. In addition, the hydrophilic ramie fibers are the main reason for the high water absorption of the composites, as they contain many easily accessible hydroxyl groups, endowing the fibers with a high level of hydrophilicity [30]. Therefore, the initial water absorption of ramie fiber/phenolic composites is high. Equilibrium water absorption is achieved, through competitive weight gain and weight loss, the moisture content remains virtually constant over time. On the one hand, RFPR undergoes chemical degradation of small resin molecules and ramie fibers under high temperatures and high-humidity conditions, such as the loss of pectin, hemicellulose and some cellulose with poor crystallization, resulting in a decrease in sample weight [31], while these damages provide extra space for water absorption, resulting in increased water absorption. On the other hand, the high moisture content of the RFRP sample (saturated moisture content is approximately 5.2%) leads to fiber swelling, leading to resin cracking or interfacial debonding between the fiber and the resin matrix, which also provides more space for the absorption of water molecules [32]. When the degradation of individual components predominated, there was a mass loss trend over time (98% humidity environment in Figure 1). The mechanism by which fibers expand with water leading to crack formation has also been reported elsewhere [33].

To compare the saturated water absorption rate and the diffusion coefficient of water molecules under various conditions, the diffusion process of water molecules in RFRP was fitted using the simplified Fick’s law equation (Equation (1)). Table 2 shows the saturated water absorption and water molecule diffusion coefficient of RFRP in different environments.

(1) $$M(t)=M_{m}\times \{1-\exp[-7.3{\left(\frac{Dt}{h^{2}}\right)}^{0.75}]\}$$

*M_m_* is the saturated water absorption, *D* is the diffusion coefficient of water molecules, *t* is the exposure time in the hot and humid environment, and *h* is the thickness of the sample.

To compare the saturated water absorption rate and the diffusion coefficient of water molecules under various conditions, the diffusion process of water molecules in RFRP was fitted using the simplified Fick’s law equation (Equation (1)). Table 2 shows the saturated water absorption and water molecule diffusion coefficient of RFRP in different environments. It shows that the saturated water content increases significantly with the increase in temperature and relative humidity.

The saturated water content (*M*_∞_) mainly depends on the relative humidity of the environment, while *D* depends on the temperature. The variation in saturated moisture content with humidity (Π) can be described by the following Equation (2):

(2) $$M_{m}=a\times \prod^{b}$$

where *M_m_* is the saturated water absorption and Π is the relative humidity.

Table 3 shows the a and b parameter values of RFRP at different temperatures and the comparison with CFRP and epoxy resin parameters [34]. According to Table 3, it can be seen that temperature has little influence on parameters a and b. The parameter a of RFRP is higher than that of CFRP and close to that of resin. The parameter b is higher than that of CFRP and resin, so the RFRP is greatly affected by humidity, and its hygroscopicity is significantly higher than that of CFRP and resin. This is because the main component of ramie fiber, cellulose, contains a large number of hydroxyl groups, and its water absorption is stronger than that of carbon fiber and resin. In addition, there are basically no gaps inside synthetic fibers such as carbon fibers [35]. The cell cavity inside the ramie fiber provides additional water storage space, and at the same time, there are many micro-gaps between the cellulose in the cell wall, and the capillary effect is very obvious [18]. The above two factors are the main reasons why the hygroscopicity of RFRP is much greater than that of CRFP.

**Figure 2:**
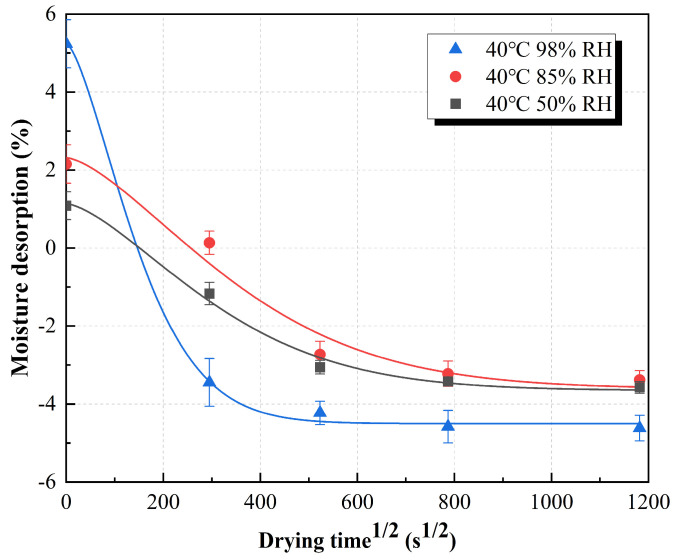
Moisture desorption curves of ramie fiber fabric–reinforced phenolic resin at 40 °C, which were exposed to three kinds of humidity at room temperature for 6 months.

After 6 months of exposure in a hydrothermal environment, the RFRP samples were subsequently dried in an oven at 60 °C, and the weight changes were recorded periodically. Determine the per cent water desorption *W*_t_ using the following Equation (3):

(3) $$W_{t}(\%)=(M_{t}-M_{0})/M_{0}\times 100$$

where *M*_t_ is the weight after drying time t and *M*_0_ is the initial weight. As shown in Figure 2, the moisture desorption results are represented by the function relationship graph of the weight loss of the sample in different humid environments and the square root of the drying time. At the same time, the water desorption process of RFRP is the inverse process of Fick’s water absorption model, and the relationship between the mass of the sample and the drying time can be fitted by Equation (4):

(4) $$m_{t}=m_{0}-m_{0}(1-\frac{1}{1+M_{d}})\{1-\exp[-7.3{\left(\frac{D_{d}t}{h^{2}}\right)}^{0.75}]\}$$

where *m*_0_ is the mass of the sample before drying, *M_d_* is the equilibrium hydrolysis adsorption amount, and *D_d_* is the diffusion coefficient of water molecules during the water adsorption process. The fitting results are shown in Figure 2. It shows that the water desorption decreases rapidly in the initial stage, and slowly reaches the leveling stage. After complete drying, the sample weight loss (indicated by negative *W*_t_ after drying) determined according to Figure 2 was approximately 3.58%, 3.17%, and 4.62% at 50% RH, 85% RH, and 98% RH, respectively. The weight loss of the samples was attributed to residual acetone (used as a diluent during prepreg preparation) and/or evaporation of small unreacted phenolic resin molecules during thermal compression. Notably, the specimens treated at the 98% RH humidity level had the greatest final weight loss. This is because when the sample is exposed to a high-humidity environment, the damage to the interior of the RFRP is more serious, and more small molecules are volatilized and dissolved in a high-temperature and high-humidity environment. Samples exposed to 85% RH and 50% RH experienced the same final weight loss.

### 3.2. Degradation of Mechanical Properties

**Figure 3:**
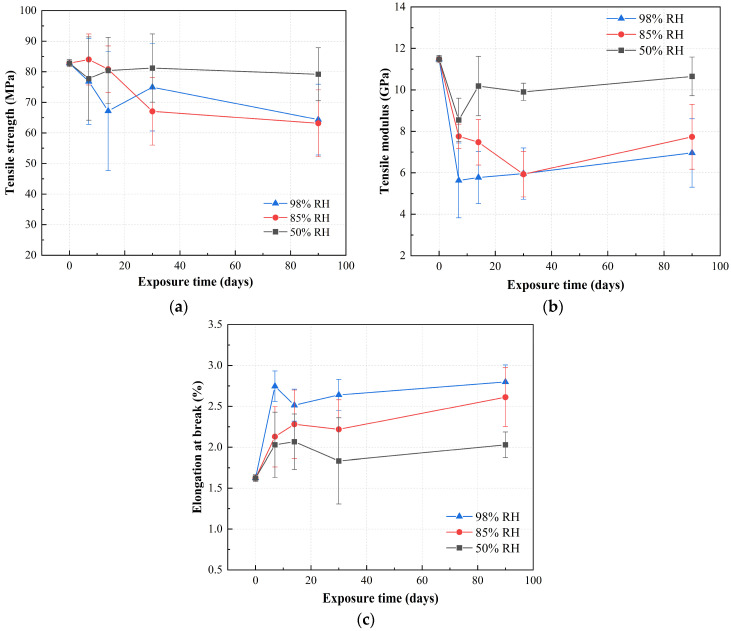
Variation in the (**a**) tensile strength, (**b**) tensile modulus, and (**c**) elongation at break as a function of exposure time of ramie fiber fabric-reinforced phenolic resin under three humidity conditions at 40 °C.

Figure 3 shows the changes in tensile properties of RFRP under three humidity environments at 40 °C. Figure 3a shows the change in tensile strength of RFRP. Under the condition of 50% RH, the tensile strength of the samples decreased slightly after the first 7 days of exposure and then recovered slowly with the prolongation of exposure time. After 90 days of exposure, the tensile strength of the RFRP sample at 50% RH was slightly lower than that of the unaged sample. The tensile strength of samples exposed to relatively high-humidity conditions (85% RH and 98% RH) decreased significantly. The influence of the two types of humidity on the tensile strength of RFRP is very similar. The tensile strength of RFRP has experienced a process of rising and falling. After 90 days of exposure, the tensile strength retention rate of RFRP in 85% RH and 98% RH environments are 76.26% and 77.71% of the original strength. During ageing, the increase in tensile strength is caused by the post-curing of the resin system [18].

Figure 3b presents the degradation of the tensile modulus of RFRP. The tensile modulus of RFRP samples is more susceptible to humidity than the tensile strength. After 7 days of exposure, the retention rates of tensile modulus of RFRP under 50% RH, 85% RH and 98% RH were 74.45%, 67.53% and 49.04%. The tensile modulus tended to increase slightly with increasing exposure time. After 90 days, the modulus retention rates of RFRP in 50% RH, 85% RH and 98% RH were 92.75%, 67.34% and 60.58%. Ramie fibers exhibit strong hydrophilic properties. Therefore, water ingress into RFRP samples can lead to the degradation of fibers, resin matrix, and fiber–matrix interface. The degradation of ramie fibers is considered to play a key role in the degradation of tensile properties, especially the degradation of tensile modulus. This trend is in contrast to conventional fibers, i.e., glass-fiber- or carbon-fiber-reinforced polymer composites, whose tensile modulus is generally not affected by hygrothermal ageing. The difference in trends can be attributed to the fact that synthetic fibers are less susceptible to moisture penetration, as opposed to ramie fibers. Ramie fiber is composed of lignin, pectin, hemicellulose, cellulose and other components, and the cellulose is wrapped in substances such as pectin. The entry of water molecules destroys the hydrogen bond between cellulose and pectin, and the modulus of ramie fiber degrades greatly. Therefore, the modulus of RFRP is greatly affected by the hot and humid environment, and the degradation is obvious.

Figure 3c shows the changes in the elongation at break of RFRP, and the elongation at break increases with the time exposed to the hydrothermal environment. After 90 days of exposure, the elongation at break increased by 24.94%, 60.78%, and 72.29% at 50% RH, 85% RH, and 98% RH, respectively. As shown in Figure 3c, the elongation at break of RFRP in hydrothermal environment first increased and then decreased. This is due to the plasticizing action of water molecules. After the water molecules enter the ramie fiber, the hemicellulose wrapped in the cellulose will be decomposed, so that the cellulose molecular chain as the main load-bearing component can move freely, and the flexibility and elongation at break will increase. With the prolongation of the exposure time, the water absorption rate further increased, causing the destruction of the cellulose and the interface, and the elongation at break decreased.

In Figure 3, the variance of the performance test results of RFRP is relatively large. This is due to the influence of the growth environment and the twisting preparation process of ramie fiber, which leads to a large performance dispersion of ramie fiber. Therefore, the performance dispersion of RFRP is relatively large, which is also the main disadvantage of natural-fiber-reinforced polymer composites.

**Figure 4:**
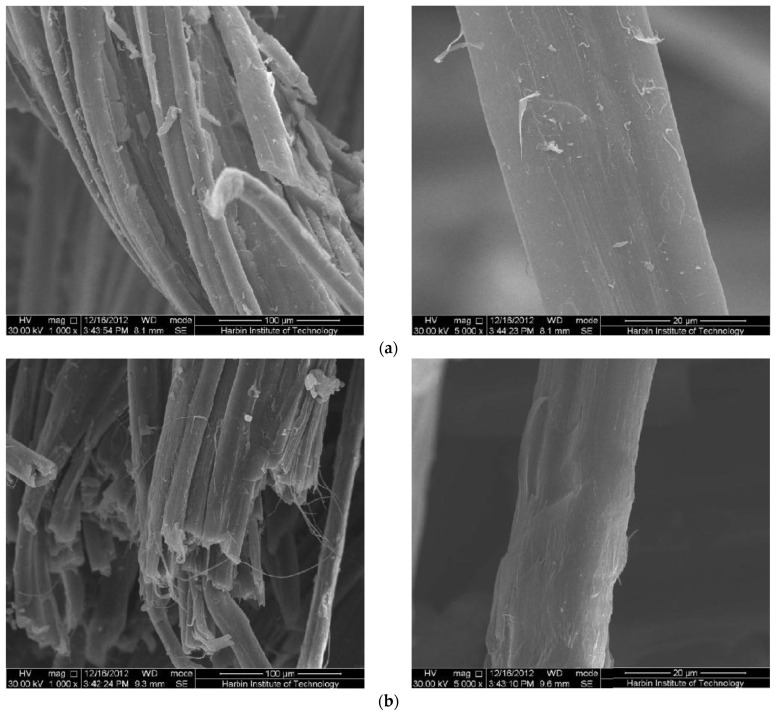
Tensile failure cross-sectional view of RFRP after ageing (exposure to 98% RH environment): (**a**) exposed for 30 days; (**b**) exposed for 90 days.

Figure 4 is the SEM photographs of the tensile section in different periods under a 98% RH environment. It can be seen from the figure that the tensile failure mode of the molded sheet is that the ramie fiber is broken and the interfacial debonded fiber is pulled out from the resin. Figure 4a is the tensile section of the sample after ageing for 30 days, and a small amount of resin is wrapped on the surface of the broken fiber. In the SEM image magnified by 5000 times, it can be seen that after being stretched longitudinally, some cracks will be produced along the fiber direction, and a small amount of peeling will appear on the skin of a single fiber. Resin debonding and falling off. Figure 4b is the tensile section of the sample after ageing for 90 days. By comparison, it can be seen that the resin content between the fibers gradually decreases with the prolongation of exposure to a hydrothermal environment, and the ramie fibers in a single bundle of fibers gradually disperse, and the resin falls off from it. This result is consistent with the previous tensile performance test results. The water absorption of the sample in a hydrothermal environment leads to fiber–resin debonding, which significantly reduces the tensile properties of the material. The SEM photos of a single fiber show that the ramie fiber swells after absorbing water, and large defects appear on the surface of the fiber due to the action of water molecules. This is similar to the degradation regularity of tensile properties obtained above. Under the action of water molecules, substances such as pectin on the fiber surface are hydrolyzed and defects appear. At the same time, the entry of water molecules caused the swelling of the ramie fiber, resulting in stress concentration at the interface between the fiber and the resin and a decrease in the interface bonding performance, so the resin content on the fiber surface on the stretched section decreased, indicating that the main failure in the stretching process is debonding of the interface and fracture of the fibers.

**Figure 5:**
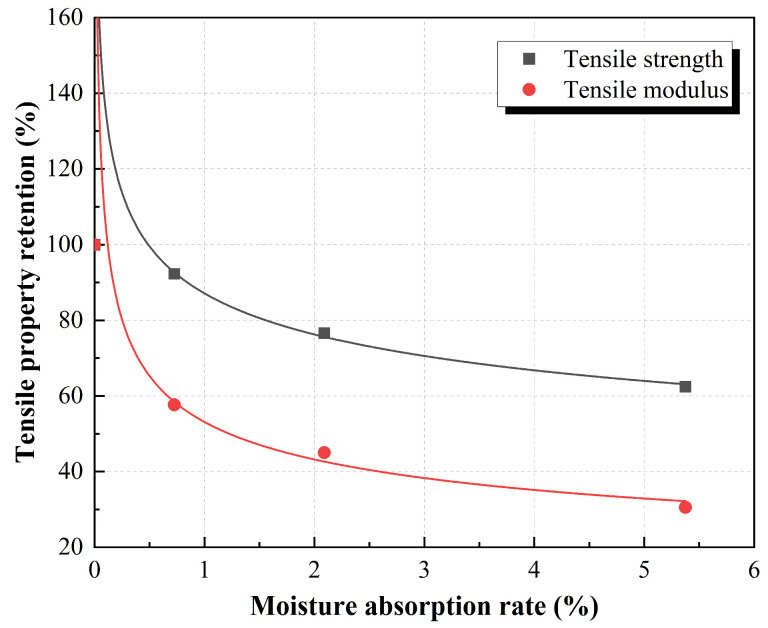
Relationship between water absorption and tensile properties retention in three months.

The root cause of the degradation of the mechanical properties of the molded sheet in a hydrothermal environment is that the material absorbs water, and water molecules enter the interior of the material, which affects the mechanical properties of the ramie fiber and phenolic resin, and more importantly, the fiber–resin interface. Therefore, it is necessary to study the relationship between water content and mechanical properties of molded sheet. The water absorption rate and the corresponding tensile properties under different hydrothermal environments at the same stage were analyzed, and a typical degradation curve was selected to obtain Figure 5, which is the change curve of tensile properties with moisture absorption rate in one month. The Equation (5) gives a relationship between moisture absorption and tensile properties.

(5) $$R=r\times M^{a}$$

where *R* is the performance retention rate and *M* is the sample saturated (equilibrium) water absorption. Among them, *r* and a are the corresponding parameters, which represent the degree of influence of moisture absorption rate on mechanical properties. By fitting and calculating the curve in Figure 5, the tensile strength influence coefficients r_1_ and a_1_ are 87% and −0.19, respectively, and the tensile modulus influence coefficients r_2_ and a_2_ are 53% and −0.29, respectively. The influence coefficient of modulus is significantly lower than that of strength, and decreases rapidly with the increase of moisture absorption.

**Figure 6:**
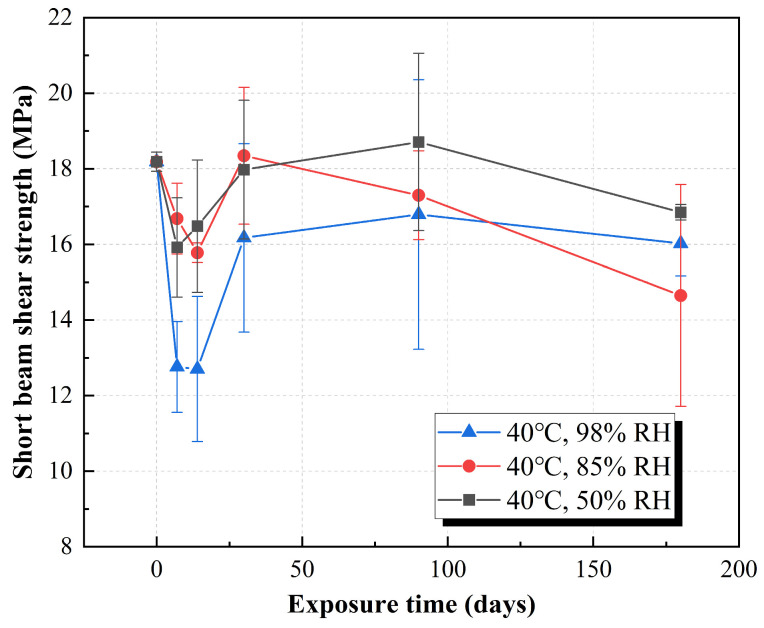
Variation in the short beam shear strength as a function of exposure time of ramie fiber fabric-reinforced phenolic resin under three humidity conditions at 40 °C.

Figure 6 is the curve of shear strength versus time of the molded sheet placed in different high-temperature and humid environments for six months. It can be seen from the figure that the shear strength decreases rapidly in the first week, and then the strength increases with a large range. After one week, the shear strength of the molded sheet decreased by 13%, 9%, and 30% under 50% RH, 85% RH and 98% RH, respectively, which indicated that the initial water absorption of the RFRP was faster. Water molecules change the fiber–resin interface in a dry state, causing the resin to swell to a certain extent to deboned the interface, and the interlayer shear strength decreases rapidly, and then the interface bonding performance is improved with the decrease of water absorption rate and post-curing of the resin. After six months, the shear strength decreased by 8%, 20%, and 12%, respectively. The shear strength characterizes the bond strength of the fiber–resin interface of the RFRP. From the perspective of the degradation trend of the shear performance of the molded sheet after absorbing water, the material tends to be flat and has a certain recovery after a large drop in the initial stage of water absorption. The impact on shear performance is not obvious, which may be due to the high water absorption rate of the material in the high-temperature environment, and the shear strength of the material drops sharply at the initial stage. Afterwards, because the equilibrium water absorption rate is determined by humidity, the decrease of shear strength increases with the increase of humidity.

**Figure 7:**
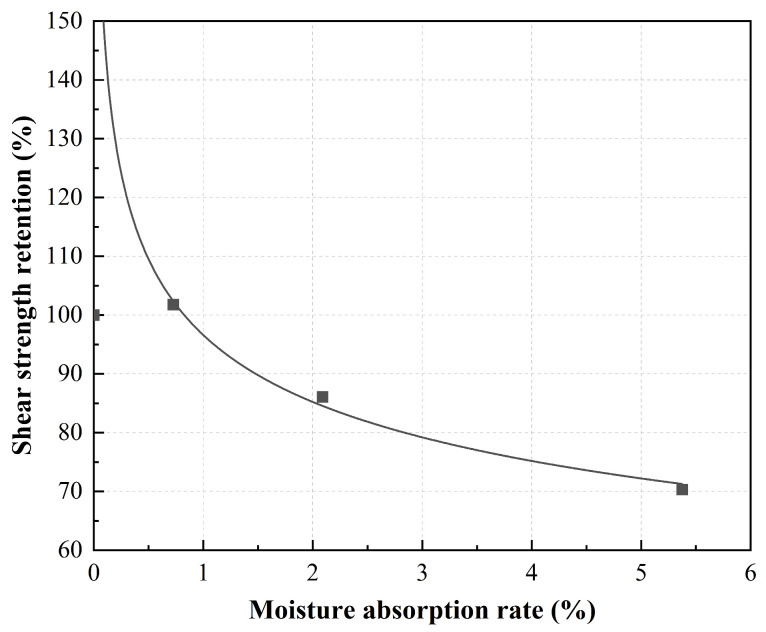
Relationship between water absorption and shear strength retention in three months.

Figure 7 shows the relationship between moisture absorption rate and shear strength for one month. The influence parameters r and a obtained by fitting are 97% and −0.18, respectively. Compared with the previous tensile influence coefficient, it can be seen that the decrease is equivalent to the tensile strength. On the other hand, it also shows that the larger water content has an adverse effect on the fiber–resin interface.

### 3.3. Mechanical Properties of Dried Samples

Table 4 shows the bending performance comparison of RFRP in dry and wet conditions. Humidity has a significant effect on the flexural strength of RFRP, that is, the flexural strength is higher before 85% RH and does not change significantly with humidity, and the flexural strength from 85% RH to 98% RH is significantly affected by humidity. The flexural modulus decreased more than the flexural strength. Humidity is the main factor affecting the degradation of RFRP flexural properties. When the material is in the bending state, the lower part is under tension, the upper part is under compression. The fiber fracture along the length direction of the lower part and the interface slip between fiber and resin cause the material to lose its bearing capacity and lead to failure. Comparing with stretching and shearing, in bending state, there is a more complex stress state inside the RFRP. As far as this experiment is concerned, after the ramie fiber absorbs moisture, the properties of the fiber itself are degraded, especially the flexibility is enhanced, and the strain is greatly increased under the same stress. In addition, due to different strain changes, debonding will inevitably occur between the fiber and resin interfaces. After water molecules enter the interior of the material, they combine with hydrogen bonds (hydrophilic groups) in the cellulose, destroying the original bond between the fiber and the resin. A bonded state, where debonding and voids occur at the interface, which has an increasing effect on the hygroscopic performance, causing more water molecules to enter the interior of the material. When the bending stress reaches a certain value, the fiber–resin interface first deboned and loses the force transmission mechanism, and the ramie fibers in the longitudinal direction provide bearing capacity, and the ramie fibers after moisture absorption are extremely flexible and the strain increases rapidly. As the stress continues to increase, it eventually leads to fiber breakage and bending failure of the RFRP.

The flexural strength of the samples exposed to hydrothermal environment recovered after drying, which was within 10% of the initial flexural strength. After drying, the flexural modulus of the sample was greater than the initial value, and the deflection also decreased. It shows that water has little effect on the flexural properties of the RFRP, and the flexural properties of the samples exposed to the hydrothermal environment for a long time recover and increase after drying, and the influence of humidity on the flexural properties recovery has no obvious regulation.

Table 5 shows the shear strength comparison of RFRP under dry and wet conditions. After 3 months of exposure in a hydrothermal environment, the shear strength decreased, and with the increase of humidity, the shear strength decreased more. After drying, the shear strength decreased further.

## 4. Summary and Conclusions

This paper studies the durability performance of ramie cloth-phenolic resin molded boards in nine different hot and humid environments, including the comparison of moisture absorption performance, mechanical properties, dry and wet weight changes and dry and wet mechanical properties of molded boards in hot and humid environments. The specific conclusions are as follows:

(1) The moisture absorption and diffusion of ramie-fiber-reinforced polymer composites (RFRP) in a hydrothermal environment conform to Fick’s law. The equilibrium moisture absorption rate is mainly determined by the ambient humidity. As the ambient humidity increases, the equilibrium moisture absorption rate increases.
(2) Mechanical properties of RFRP in a hydrothermal environment decline quickly in the first week, and then tend to be flat, and the mechanical properties of the material decrease significantly with the increase in humidity.
(3) The water molecules reduce the interfacial bonding performance, which leads to the degradation of the mechanical properties of the ramie-fiber-reinforced composites in the hydrothermal environment, especially the significant decrease of the modulus.
(4) After RFRP was treated in a hydrothermal environment, its weight loss was between 2.5% and 4.5%, and it increased with the increase in humidity. After drying, the mechanical properties of the RFRP significantly recover, similar to the initial value, and water has an irreversible effect on RFRP.

## Figures and Tables

**Table 1:** Humidity corresponding to different saturated salt solutions under 40 °C.

Saline Solution	Solubility at Room Temperature (g/mL)	Humidity (RH)
NaBr	0.903	50%
KCl	0.357	85%
K_2_SO_4_	0.120	98%

**Table 2:** The maximum moisture absorption and diffusion coefficient (*D*) of ramie fiber fabric-reinforced phenolic resin samples under three kinds of humidity in 90 days.

Hydrothermal Environment	Saturated Water Absorption *M*_m_ (%)	Diffusion Coefficient *D* (×10^−6^ mm^2^/s)
40 °C 50% RH	1.01	0.95
40 °C 85% RH	2.31	2.52
40 °C 98% RH	5.24	1.45

**Table 3:** The a and b values of ramie fiber fabric-reinforced phenolic resin composites under two temperature conditions and the comparison with CFRP and epoxy resin [34].

Materials	a	b
RFRP	0.06	4.93
CFRP	0.01~0.02	1
Resin	0.04~0.07	1~2

**Table 4:** Flexural performance comparison under dry and wet conditions.

Properties	Humidity Environments	Control	Standard Deviations	Three Monthes	Rate of Change	Drying	Rate of Change
Strength	50% RH	135.24 MPa	9.87 MPa	106.69 MPa	−21.11%	121.56 MPa	−10.12%
	85% RH	135.24 MPa	9.87 MPa	106.05 MPa	−21.58%	133.11 MPa	−1.57%
	95% RH	135.24 MPa	9.87 MPa	119.14 MPa	−11.90%	141.46 MPa	4.60%
Modulus	50% RH	9.69 GPa	0.82 GPa	7.71 GPa	−20.43%	10.39 GPa	7.22%
	85% RH	9.69 GPa	0.82 GPa	6.22 GPa	−35.81%	11.7 GPa	20.74%
	95% RH	9.69 GPa	0.82 GPa	5.47 GPa	−43.55%	11.28 GPa	16.41%
Deflection	50% RH	3.05 mm	0.52 mm	3.75 mm	22.95%	1.71 mm	−43.93%
	85% RH	3.05 mm	0.52 mm	5.02 mm	64.59%	1.86 mm	−39.02%
	95% RH	3.05 mm	0.52 mm	4.14 mm	35.74%	2.15 mm	−29.51%

**Table 5:** Comparison of shear strength under dry and wet conditions.

Humidity Environments	Control	Standard Deviations	Three Months	Rate of Change	Drying	Rate of Change
50% RH	18.26 MPa	0.25 MPa	18.70 MPa	2.41%	14.73 MPa	−19.33%
85% RH	18.26 MPa	0.25 MPa	17.30 MPa	−5.26%	13.36 MPa	−26.83%
95% RH	18.26 MPa	0.25 MPa	16.80 MPa	−8.00%	15.61 MPa	−14.51%

## Data Availability

Data are available from the authors on request.
